# Effects of short‐term endurance exercise on gut microbiota in elderly men

**DOI:** 10.14814/phy2.13935

**Published:** 2018-12-08

**Authors:** Hirokazu Taniguchi, Kumpei Tanisawa, Xiaomin Sun, Takafumi Kubo, Yuri Hoshino, Masahito Hosokawa, Haruko Takeyama, Mitsuru Higuchi

**Affiliations:** ^1^ Division of Applied Life Sciences Graduate School of Life and Environmental Sciences Kyoto Prefectural University Kyoto Japan; ^2^ Department of Physical Activity Research National Institutes of Biomedical Innovation Health and Nutrition Tokyo Japan; ^3^ Research Fellow of Japan Society for the Promotion of Science Tokyo Japan; ^4^ Institute of Advanced Active Aging Research Tokorozawa Japan; ^5^ Department of Nutrition and Food Safety School of Public Health Xi’ an Jiaotong University Xi'an China; ^6^ Global Health Institute Xi'an Jiaotong University Health Science Center Xi'an China; ^7^ Faculty of Sport Sciences Waseda University Tokorozawa Japan; ^8^ Graduate School of Sport Sciences Waseda University Tokorozawa Japan; ^9^ Department of Life Science and Medical Bioscience Waseda University Tokyo Japan

**Keywords:** Cardiovascular health, *Clostridium difficile*, microbiome, *Oscillospira*, PICRUSt

## Abstract

Regular exercise reduces the risks for cardiovascular diseases. Although the gut microbiota has been associated with fitness level and cardiometabolic risk factors, the effects of exercise‐induced gut microbiota changes in elderly individuals are unclear. This study evaluated whether endurance exercise modulates the gut microbiota in elderly subjects, and whether these changes are associated with host cardiometabolic phenotypes. In a randomized crossover trial, 33 elderly Japanese men participated in a 5‐week endurance exercise program. 16S rRNA gene‐based metagenomic analyses revealed that the effect of endurance exercise on gut microbiota diversity was not greater than interindividual differences, whereas changes in *α*‐diversity indices during intervention were negatively correlated with changes in systolic and diastolic blood pressure, especially during exercise. Microbial composition analyses showed that the relative abundance of *Clostridium difficile* significantly decreased, whereas that of *Oscillospira* significantly increased during exercise as compared to the control period. The changes in these taxa were correlated with the changes in several cardiometabolic risk factors. The findings indicate that short‐term endurance exercise has little effect on gut microbiota in elderly individuals, and that the changes in gut microbiota were associated with cardiometabolic risk factors, such as systolic and diastolic blood pressure, providing preliminary insight into the associations between the gut microbiota and cardiometabolic phenotypes.

## Introduction

Specific features of the gut microbiome, including bacterial species composition and diversity, may be associated with metabolic disorders, such as obesity (Turnbaugh et al. 2009; Le Chatelier et al. 2013) and type 2 diabetes (Larsen et al. 2010; Qin et al. 2012). Studies on the human gut microbiota have revealed considerable interindividual differences, even in healthy populations (Human Microbiome Project C, 2012). The gut microbiota is influenced by various factors, including diet (Chen et al. 2014; Voreades et al. 2014), host genetics (Turnbaugh et al. 2010; Goodrich et al. 2014), and the environment (Costello et al. 2009; Yatsunenko et al. 2012). Due to the large interindividual differences in gut microbiota and its associated factors, the relationship between the gut microbiome and host metabolism is very complex.

The effect of physical fitness on the gut microbiota is a popular topic of investigation. Recent animal studies have demonstrated that endurance exercises can help modulate the composition and diversity of the gut microbiota (Evans et al. 2014; Denou et al. 2016). Human cross‐sectional studies have also shown that the gut microbiota of professional rugby athletes is more diverse as compared with that of control subjects (Clarke et al. 2014). In addition, cardiorespiratory fitness (CRF) level has been positively correlated with gut microbiota diversity in healthy participants (Estaki et al. 2016). Moreover, recent studies demonstrated the association of the frailty index, which mainly assesses physical function, with gut microbiota composition (Claesson et al. 2012) and bacterial diversity (Jackson et al. 2016) in the elderly. Therefore, it is possible that exercise has important roles also in the formation of the human gut microbiota.

Older adults typically show higher blood pressure, which is a major risk factor for cardiovascular events (Lewington et al. 2002). Although the underlying mechanisms have not been fully elucidated, it has been well‐documented that increased CRF due to regular exercise reduces the risks for hypertension (Cornelissen and Fagard 2005) and cardiovascular diseases (Kodama et al. 2009). In addition, some animal studies revealed that gut microbiota plays a key role in hypertension (Adnan et al. 2017; Santisteban et al. 2017), and recent human studies have also reported that the gut microbiota is less diverse in hypertensive patients (Yang et al. 2015; Li et al. 2017). Age‐related changes in gut microbiota diversity and composition have been suggested (Yatsunenko et al. 2012; O'Toole and Jeffery 2015). Thus, we hypothesized that the gut microbiota is associated with hypertension with age, and may be improved by increased CRF.

Therefore, exercise interventions, particularly in older adults, can provide key insights into the associations between the gut microbiome, exercise, and cardiometabolic risks. The current study aimed to evaluate whether endurance exercise can modulate the gut microbiota in elderly subjects, and whether exercise‐induced changes in the gut microbiota were associated with host cardiometabolic phenotypes.

## Materials and Methods

### Ethics statement

All experimental protocols were approved by the Ethics Committee of Waseda University, and written informed consent was obtained from all participants prior to their enrollment into the study. The study was conducted in accordance with the principles outlined in the Declaration of Helsinki, and is registered in UMIN‐Clinical Trial Registry (number: UMIN000018374; registration data: 22/07/2015) to investigate the effects of endurance exercise on peptide hormones and the gut microbiota. The peptide hormone fibroblast growth factor 21 (FGF21) was significantly reduced by the 5‐week endurance exercise program, as described previously (Taniguchi et al. 2016).

### Subjects

Healthy elderly Japanese individuals aged over 60 years were recruited by temporary employment agencies. We included subjects who had maintained a consistent lifestyle, diet, and body weight in the most recent decade. We excluded individuals with no previous history of gastrointestinal disorder as well as those that avoided the use of pre‐, pro‐, syn‐, and antibiotics prior to entering the study.

Thirty‐three elderly Japanese men (aged 62–76 years) were recruited from the local community in the Tokorozawa metropolitan area. The individuals had not previously participated in chronic nutritional and/or exercise studies. None of the subjects had been diagnosed with diabetes, cardiovascular diseases, cancer, chronic kidney diseases, and autoimmune disorders. The clinical history of the subjects is shown in Table 1. Two subjects had high levels of fasting glucose (≥126 mg/dL) at baseline. Six subjects (18.2%) were taking antihypertensive drugs, and four subjects (12.1%) were using lipid‐lowering medication. The medication status was not changed, and the elderly individuals were instructed to avoid any drugs affecting gastrointestinal stimulation for the duration of the study.

**Table 1:** Clinical history of the subjects (*N* = 33)

Disease	*N*	%
Hypertension	6	18.2
Dyslipidemia	4	12.1
Hyperuricemia	2	6.1
Prostatic hyperplasia	2	6.1

### Study design

**Figure 1:**
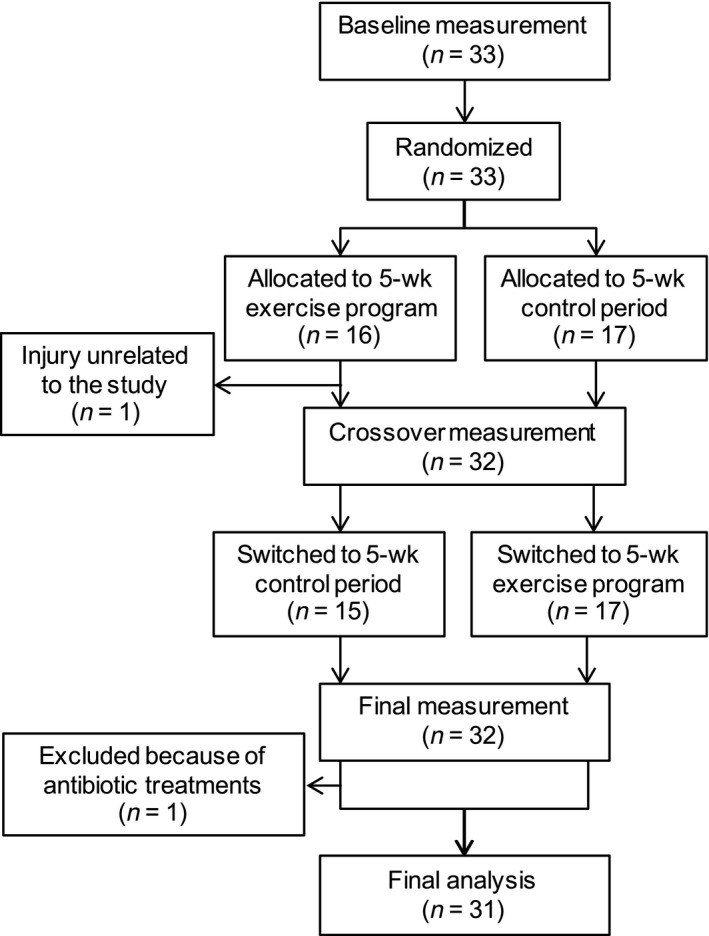
Flow diagram of the randomized crossover trial.

A randomized crossover trial design was used to compare intra‐individual differences during control and exercise sessions, since large interindividual differences in human gut microbiota have been observed (Human Microbiome Project C, 2012). The study design was detailed previously (Taniguchi et al. 2016). Briefly, the subjects were randomly allocated to either a 5‐week endurance exercise program or a control period. After 5 weeks, the subjects switched to the other regimen for another 5 weeks (Fig. 1). A randomization was performed by a third party who was not involved in this study. All other daily physical activity levels assessed in a questionnaire remained unchanged throughout the intervention (Taniguchi et al. 2016).

### Cardiorespiratory fitness

CRF was assessed using a maximal‐graded exercise test using a cycle ergometer (Aerobike 75XLII; Combi Wellness, Inc., Tokyo, Japan) and was quantified as the peak oxygen uptake ($\dot{V}O_{2}\text{peak}$) as previously described (Taniguchi et al. 2016). The graded cycle exercise began at a workload of 40–60 W, which was then increased by 30 W every 3 min until exhaustion. This incremental protocol was used to assess the $\dot{V}O_{2}\text{peak}$ for elderly individuals (Martinez‐Caro et al. 1984). Each participant's expired gas was collected and the O_2_ and CO_2_ concentrations were measured. These concentrations were averaged over 30‐sec intervals using an automated gas analysis system (Aeromonitor AE‐310S; Minato Medical Science, Osaka, Japan). Heart rate was monitored by electrocardiography (BSM‐2401, Nihon Kohden, Tokyo, Japan) throughout the exercise period. Ratings of perceived exertion were recorded each minute during exercise. The highest value of $\dot{V}O_{2}$ recorded during the exercise test was considered the $\dot{V}O_{2}$ peak (mL/kg/min), and achievement of $\dot{V}O_{2}\text{peak}$ was accepted if at least three of the following four criteria were achieved: the $\dot{V}O_{2}$ curve showed a plateau despite increasing the work rate, the maximal heart rate was 95% of the age‐predicted maximal heart rate (220 – age in years), the respiratory exchange ratio was greater than 1.1, and the subject achieved a rating of perceived exertion of 18 or greater.

### Dietary and nutritional intake

Diet and nutritional intake were assessed via a brief (four‐page) self‐administered diet history questionnaire. The questionnaire collected information on the consumption frequency of selected foods to estimate the dietary intake of 58 food and beverage items (Kobayashi et al. 2012). The validity of the questionnaire to assess nutrient intake was confirmed using semiweighed 16‐day dietary records as the reference (Kobayashi et al. 2012).

### Endurance exercise program

Participants undertook a supervised, progressive, 5‐week aerobic exercise program, which met the current guidelines for adult prescribed exercise (Garber et al. 2011). Exercises comprised three cycle ergometer sessions per week. Exercise intensity was gradually increased such that subjects exercised at a power output designed to elicit 60% of preexercise $\dot{V}O_{2}\text{peak}$ during week 1, 70% during weeks 2 and 3, and 75% during weeks 4 and 5. The exercise duration was 30 min for weeks 1 and 2, and 45 min for weeks 3–5. We recorded the heart rate during every training session using electrocardiography (BSM‐2401; Nihon Kohden, Tokyo, Japan) to confirm that the participants exercised at their target intensities. The mean heart rate and percentage of maximal heart rate (159 ± 18 beat/min) during the exercise sessions were 116 beats/min and 73.0% for week 1, 130 beats/min and 81.8% for week 2–3, and 139 beats/min and 87.4% for week 4–5, respectively, which corresponded to exercise intensities of 55% $\dot{V}O_{2}\text{peak}$, 69% $\dot{V}O_{2}\text{peak}$, and 77% $\dot{V}O_{2}\text{peak}$, respectively (Swain et al. 1994).

### Magnetic resonance spectroscopy and imaging

The previously described (Taniguchi et al. 2016) intrahepatic ^1^H magnetic resonance spectroscopy was performed using a 1.5 T whole‐body scanner (Signa 1.5 T; General Electric, Inc., Boston, MA, USA) with 8‐channel body array coils. Data were quantified using the LCModel software version 6.3. The coefficient of variation between measurements was 2.0%. Visceral and subcutaneous fat areas were also measured by magnetic resonance imaging. The coefficient of variation for the cross‐sectional area at the umbilical level was 0.4%.

### Blood sampling and analysis

Blood samples were collected following an overnight fast. Samples were centrifuged at 3000 rpm at 4°C for 15 min. Serum and plasma were collected and stored at −80°C until analysis. The serum enzymatic activities of aspartate aminotransferase (AST), alanine aminotransferase (ALT), and gamma‐glutamyl transferase (*γ*‐GTP), as well as the concentrations of triglycerides, fasting glucose, glycated hemoglobin (HbA1c), and fasting insulin were determined by a contracted company (BML, Inc., Tokyo, Japan).

### Blood pressure

Brachial systolic blood pressure (SBP) and diastolic blood pressure (DBP) were measured using the oscillometric method (HEM‐7122; OMRON, Inc, Kyoto, Japan). Measurements were made in duplicate with participants at rest in a sitting position.

### Cardio‐ankle vascular index

To assess arterial stiffness, we measured the cardio‐ankle vascular index (CAVI) (Shirai et al. 2006) using the VaSera VS‐1500 system (Fukuda Denshi, Tokyo, Japan), as previously described (Kawano et al. 2012). The day‐to‐day coefficient of variation in the CAVI was 2 ± 1%.

### Fecal sample collection

Fecal samples were collected from subjects using commercial containers (TechnoSuruga Laboratory Co., Ltd., Shizuoka, Japan) containing guanidine thiocyanate (GuSCN) solution (100 mmol/L Tris‐HCl [pH 9.0], 40 mmol/L Tris‐EDTA [pH 8.0], 4.0 mol/L GuSCN, and 0.001% Bromothymol blue) that inhibits bacterial growth via protein denaturation. Samples were stored at 4°C until DNA extraction.

### Gut microbial DNA extraction

Gut microbial DNA was extracted from fecal samples within a month of sample collection. DNA extraction assays were performed by TechnoSuruga Laboratory Co., Ltd. (Shizuoka, Japan) as described previously (Hisada et al. 2015). Fecal solids in suspension were broken down using the FastPrep 24 Instrument (MP Biomedicals, Santa Ana, CA, USA) with zirconia beads at 5 m/sec for 2 min. DNA was extracted from a 200‐mL suspension using the Magtration System 12GC (Precision System Science, Chiba, Japan). The MagDEA^®^ DNA 200 kit (Precision System Science) was used for automatic nucleic acid extraction. Extracted DNA was stored at 4°C until sequencing was done.

### 16S rRNA sequencing and quality control

V3–V4 hypervariable regions of the 16S rRNA gene were amplified by PCR using the following primers:

341F‐5′‐TCGTCGGCAGCGTCAGATGTGTATAAGAGACAGCCTACGGGNGGCWGCAG‐3′

806R‐5′‐GTCTCGTGGGCTCGGAGATGTGTATAAGAGACAGGACTACHVGGGTATCTAATCC‐3′

PCR amplification was carried out in a 25 *μ*L reaction mixture containing 5.0 *μ*L primer (1.0 *μ*mol/L), 12.5 *μ*L 2 × KAPA HiFi HotStart Ready Mix (Kapa Biosystems, Wilmington, MA, USA), and 2.5 *μ*L microbial DNA (5 ng/*μ*L). The cycling parameters were as follows: one denaturation cycle at 95°C for 3 min, followed by 25 cycles consisting of denaturation at 95°C for 30 sec, annealing at 55°C for 30 sec, and extension at 72°C for 30 sec. A final extension was carried out at 72°C for 5 min. PCR amplicon size was confirmed by agarose gel electrophoresis. Amplified products were purified using the AMPure XP system (Beckman Coulter, Inc., Sacramento, CA, USA), according to the manufacturer's instructions.

To perform multiplex sequencing, adapters and barcodes were attached to amplicons using the Nextera XT Index Kit v2 (Illumina Inc., San Diego, CA, USA). Index PCR was carried out in 50 *μ*L reaction mixtures containing 5.0 *μ*L of each primer, 25.0 *μ*L 2 × KAPA HiFi HotStart Ready Mix, and 5.0 *μ*L PCR product. The amplification conditions were as follows: one denaturation cycle at 95°C for 3 min; eight cycles consisting of denaturation at 95°C for 30 sec, annealing at 55°C for 30 sec, and extension at 72°C for 30 sec; followed by a final extension at 72°C for 5 min. Barcoded amplicons were purified using the AMPure XP system (Beckman Coulter). The quality of the fragmentation and purification processes was assessed using an Agilent 2100 Bioanalyzer with a DNA1000 kit (Agilent Technologies, Santa Clara, CA, USA). The DNA library was diluted to a final concentration of 4 nmol/L.

The DNA library was sequenced using the Illumina MiSeq 2 × 300 bp platform with the Miseq reagent kit v3 (Illumina Inc.), according to the manufacturer's instructions. The threeˈ region of sequences with a Phred quality score less than 30 and Illumina adaptor sequences were trimmed, and then average quality score of less than 25 and read length of less than 150 bp were removed using Trimmomatic ver 0.36 (Bolger et al. 2014). Trimmed and quality‐filtered paired‐end reads were merged using the fastq‐join command in the ea‐utils software package (Expression Analysis, Durham, NC, USA).

### Operational taxonomic unit clustering and taxonomic assignment

Merged sequence reads of each sample were de‐multiplexed and the chimeric sequences were then removed using USEARCH v6.1 within the Quantitative Insights Into Microbial Ecology (QIIME) software package version 1.9.1 (Caporaso et al. 2010). Quality‐filtered sequence reads were assigned to operational taxonomic units (OTUs) using closed‐reference OTU picking at 97% identity with the UCLUST algorithm (Edgar 2010) and were compared against reference sequence collections in the Greengenes database (August 2013 release). Taxonomic assignment of the reference sequence was used for each OTU using the QIIME pipeline, and taxonomy tables at the phylum to species levels were generated. We restricted the analyses to core gut microbiome, out of which sequence reads were detected in more than 80% of the samples throughout the intervention. The OTU table of core gut microbiome was generated by compute_core_microbiome.py in QIIME.

In OTU clustering and taxonomic assignment, a total of 8,551,896 sequence reads were obtained from 93 fecal samples provided by 31 subjects at baseline, 5 weeks, and 10 weeks. On average, 91,956 ± 43,466 reads were obtained per sample, which were assigned to 6471 OTUs. Sequence reads corresponding to 15 phyla, 26 classes, 52 orders, 108 families, 255 genera, and 363 species were detected in the fecal samples.

### Diversity analyses

Diversity analyses were conducted with a rarefied OTU table containing 17,846 sequence reads, which was consistent with the minimum read number of the analyzed samples. QIIME software was used. To assess the gut microbiota diversity within each sample, we calculated *α*‐diversity indices, such as the Shannon diversity index (Shannon 1948) and the observed species (observed OTUs). To assess gut microbial communities at the OTU level, the *β*‐diversity indices were calculated on rarefied sequence reads based on weighted and unweighted Unifrac distances (Lozupone and Knight 2005) and Bray‐Curtis dissimilarity matrices (Bray and Curtis 1957). Principal coordinate analysis (PCoA) was also performed based on these distance/dissimilarity matrices, and PCoA plots were used to visualize data similarities.

### Functional metagenome prediction

We used phylogenetic investigation of communities by reconstruction of unobserved states (PICRUSt) to predict functional metagenomic profiles from 16S rRNA gene sequences (Langille et al. 2013). The genus level OTU table rarefied to 17,846 reads were normalized to adjust for different 16S rRNA gene copy numbers, and functional genes were predicted based on the Kyoto Encyclopedia of Genes and Genomes (KEGG) database (Ogata et al. 1999).

### Statistical analyses

All statistical analyses were performed using SPSS version 23.0 software (SPSS, Inc, Chicago, IL, USA), R version 3.4.3 (Team 2017), or QIIME version 1.9.1. Since we could not predict how much the 5‐week exercise period would affect the relative abundance of each taxa and diversity index, we calculated the sample size based on the expected changes in the $\dot{V}O_{2}\text{peak}$. We expected a 10% (2.5 mL/kg/min) increase in the $\dot{V}O_{2}\text{peak}$ in participants with a $\dot{V}O_{2}\text{peak}$ of 25 mL/kg/min by the end of the 5‐week exercise period, assuming a standard deviation of 2.5 mL/kg/min. A sample size of 27 was calculated to achieve a verification power of 95% with a significance level of *α* = 0.05. We also calculated the post hoc statistical power for the variables with significant changes between the exercise and control periods. The Kolmogorov‐Smirnov test was performed to assess the normality of the data distribution. Since we used a 2 × 2 crossover study design, we analyzed the treatment, carryover, and period effects by using the unpaired Student's *t*‐test (for normally distributed variables) or the Mann–Whitney *U* test (for nonnormally distributed variables) according to the standard statistical methods for the analysis of crossover trials (Jones and Kenward 2014). If a significant treatment effect was detected from the gut microbiota data, we further performed an analysis of covariance including potential confounders affecting gut microbiota composition as appropriate. Differences in physical activity level and dietary and nutritional intake at baseline, 5 weeks, and 10 weeks were assessed by repeated one‐way ANOVA, followed by post hoc analysis via the paired Student's *t*‐test for normally distributed data. For nonnormally distributed data, the Friedman's test was carried out, followed by the Wilcoxon signed‐rank test with Bonferroni correction. Associations between changes in all variables during exercise and control periods were determined by Spearman's rank correlation coefficients. Permutational multivariate analysis of variance (PERMANOVA) was used to assess the changes in the gut microbial communities between the exercise and nonexercise time periods (McArdle and Anderson 2001). PERMANOVA was performed bycompare_categories.py in QIIME. All measurements and calculated values were presented as mean ± standard deviation or medians (interquartile range). The level of statistical significance was set at *P* < 0.05. Benjamini‐Hochberg multiple testing adjustment was applied for all taxonomic data or predicted metagenomic data, and a cut‐off value of false discovery rate (FDR) set at 0.3.

### Data availability

All paired‐end 16S rRNA gene sequencing reads were deposited into the DDBJ database with the accession number DRA005883.

## Results

### Changes in exercise‐ and gut microbiota‐associated variables during exercise intervention

A randomized crossover trial involving 33 elderly Japanese men was designed to compare intra‐individual differences during exercise and control sessions. The subjects were randomly allocated to either a 5‐week endurance exercise program or a control period, followed by a switch to the other regimen for another 5 weeks (Fig. 1). One subject did not complete the exercise program as he had an injury unrelated to the study. Another subject was also excluded from the analyses because abnormal changes (e.g., relative abundance of *Bacteroides* changed from 0.34 to 0.03 in the control period) in several taxa were observed, which may have been due to antibiotic use. Therefore, complete data were obtained from 31 participants.

Changes in measured variables during the 5‐week endurance exercise program and the control period are summarized in Table 2. Since we used a 2 × 2 crossover design, we examined the treatment, carryover, and period effects to compare the changes of variables between exercise and control periods, as well as to examine the effect of the sequence of regimen on the changes in the variables, according to standard statistical methods for the analysis of crossover trials (Jones and Kenward 2014). $\dot{V}O_{2}\text{peak}$ and high density lipoprotein (HDL) cholesterol levels significantly increased during the exercise period as compared to the control period. We did not detect significant carryover effects on these variables. Total cholesterol level was also increased during the exercise period as compared to the control period, but carryover effects tended to be significant. In contrast, CAVI, intrahepatic fat content, and levels of HbA1c significantly decreased during the exercise period when compared to the control period. Significant carryover effects were not detected in these variables.

**Table 2:** Changes in the variables during 5‐week control and exercise periods

Variables	Control			Exercise			*P* treatment	*P* carryover	*P* period
	Pre	Post	∆	Pre	Post	∆			
Body weight (kg)	63.6 ± 8.9	63.6 ± 8.6	0.0 ± 1.0	63.6 ± 8.7	63.6 ± 9.0	0.0 ± 0.8	0.931	0.698	0.046
BMI (kg/m^2^)	22.9 ± 2.5	22.9 ± 2.4	0.0 ± 0.4	22.9 ± 2.5	22.9 ± 2.6	0.0 ± 0.4	0.752	0.663	0.624
Body fat (%)	20.6 ± 5.1	20.9 ± 5.2	0.3 ± 2.0	21.0 ± 5.0	20.5 ± 4.7	−0.5 ± 2.7	0.336	0.933	0.495
SBP (mmHg)	144.1 ± 22.4	142.1 ± 21.2	−2.0 ± 15.3	148.9 ± 21.8	139.9 ± 21.2	−9.0 ± 14.4	0.173	0.121	0.715
DBP (mmHg)	86.5 ± 9.4	82.7 ± 8.9	−3.8 ± 8.8	86.6 ± 11.3	83.5 ± 10.4	−3.0 ± 8.3	0.845	0.373	0.211
CAVI	8.36 ± 0.95	8.46 ± 1.02	0.10 ± 0.50	8.55 ± 1.04	8.26 ± 0.99	−0.29 ± 0.60	**0.019**	0.851	0.135
$\dot{V}O_{2}\text{peak}$ (mL/kg/min)*	26.0 ± 5.3	25.2 ± 4.4	−0.8 ± 2.9	24.5±4.1	27.3 ± 4.6	2.8 ± 2.7	**<0.001**	0.230	0.102
Visceral fat area (cm^2^)	99.7 ± 42.7	101.3 ± 44.4	1.6 ± 10.0	100.4±42.6	99.8 ± 43.3	−0.6 ± 12.8	0.532	0.705	0.754
Subcutaneous fat area (cm^2^)	113.2 ± 41.5	110.1 ± 37.4	−3.1 ± 14.8	111.6±37.2	112.5 ± 42.5	0.9 ± 20.2	0.395	0.750	0.131
Intrahepatic fat (%)	3.9 ± 3.1	4.2 ± 3.6	0.3 ± 1.3	4.1 ± 3.5	3.6 ± 3.0	−0.5 ± 1.2	**0.046**	0.317	0.929
AST (IU/L)*	26.4 ± 8.8	27.8 ± 10.3	1.4 ± 4.6	28.3 ± 14.2	26.3 ± 8.9	−2.0 ± 7.8	0.092	0.625	0.058
ALT (IU/L)*	22.5 ± 9.9	22.5 ± 10.5	−0.1 ± 5.5	23.2 ± 11.9	21.0 ± 9.2	−2.2 ± 5.9	0.468	0.769	**0.001**
*γ*‐GTP (IU/L)*	50.6 ± 82.5	52.4 ± 99.1	1.8 ± 17.9	56.0 ± 107.7	48.1 ± 82.5	−7.9 ± 29.1	0.444	0.518	**0.029**
Total cholesterol (mg/dL)	204.4 ± 29.7	195.0 ± 28.9	−9.4 ± 17.8	201.3 ± 31.7	204.1 ± 32.0	2.8 ± 17.6	**0.018**	0.064	0.277
HDL cholesterol (mg/dL)	61.6 ± 15.1	58.5 ± 13.5	−3.1 ± 6.8	59.5 ± 13.0	61.0 ± 14.8	1.5 ± 6.5	**0.019**	0.599	0.081
LDL cholesterol (mg/dL)	117.5 ± 28.7	110.3 ± 29.9	−7.2 ± 15.7	114.6 ± 31.8	116.1 ± 29.8	1.5 ± 13.7	0.051	0.174	0.125
Triglyceride (mg/dL)*	115.3 ± 65.9	116.1 ± 76.8	0.8 ± 57.0	121.0 ± 76.4	111.4 ± 69.5	−6.8 ± 71.3	0.830	0.399	0.949
Fasting glucose (mg/dL)	98.1 ± 13.0	99.4 ± 12.8	1.3 ± 6.2	100.3 ± 12.3	99.5 ± 11.0	−0.7 ± 6.6	0.328	0.107	**0.004**
HbA1c (%)*	5.43 ± 0.40	5.47 ± 0.41	0.05 ± 0.14	5.46 ± 0.41	5.41 ± 0.39	−0.06 ± 0.16	**0.032**	0.297	**<0.001**
Fasting insulin (*μ*U/mL)	6.9 ± 4.4	6.0 ± 4.2	−0.9 ± 2.3	6.8 ± 4.2	6.9 ± 4.3	0.1 ± 3.0	0.242	0.063	0.545

Data are mean ± SD values. Boldface indicates significance (*P* < 0.05). *P*‐values were obtained by unpaired Student's *t*‐test or *Mann–Whitney *U*‐test.

We assessed the changes in dietary intake during exercise intervention because it might affect the diversity and composition of gut microbiota. Light‐colored vegetable intake at 10 weeks was higher than that at baseline (133 [97–172] vs. 163 [109–208], *P* = 0.015). Seaweed and rice intake at 10 weeks were also higher than that at 5 weeks (Seaweed: 10.1 [5.7–15.7] vs. 15.1 [6.3–15.7], *P* = 0.004; rice: 262 [135–300] vs. 312 [150–450], *P* = 0.01). While changes in food intake may potentially affect gut microbiota due to changes in nutrition (e.g., fiber and micronutrients), we confirmed that the changes in food intake were comparable between the exercise and control periods (data not shown), and that the nutritional intake was not significantly altered during the exercise intervention (data not shown).

### Gut microbial communities display stable interindividual variations even during exercise intervention

The mean values of *α*‐diversity indices including Shannon diversity index and observed OTUs were 5.65 ± 0.66 and 545 ± 126, respectively. We compared the changes in these *α*‐diversity indices before and after the 5‐week endurance exercise regimen. None of the changes in *α*‐diversity indices were different between the exercise and control periods (data not shown).

**Figure 2:**
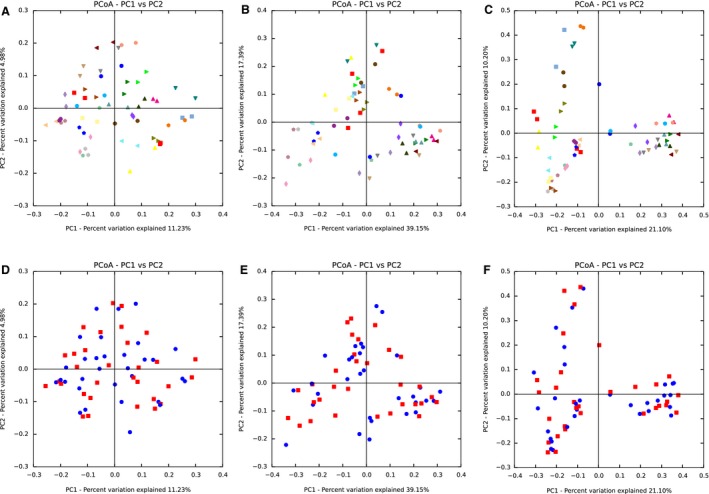
Gut microbial communities during exercise and control periods. Images represent individual principal coordinate analysis plots showing gut microbial communities at the operational taxonomic unit level before and after the exercise intervention based on (A and D) unweighted UniFrac distance, (B and E) weighted UniFrac distance, and (C and F) Bray‐Curtis dissimilarity matrices. In the upper figures (A–C), same colors on plots indicate the same individual before and after exercise. In the lower figures (D–F), blue plots represent individuals before exercise and red plots represent individuals after exercise.

We next calculated the *β*‐diversity metrics, which indicate dissimilarities in bacterial communities among samples, based on the sequence reads from pre‐ and postexercise samples to evaluate changes in the gut microbial communities caused by the 5‐week endurance exercise regimen. PCoA plots showing gut microbial communities at the OTU level were generated based on the weighted and unweighted Unifrac distances, as well as the Bray–Curtis dissimilarity matrices. The plots indicated that the gut microbial communities were almost identical between the exercise and nonexercise time periods (PERMANOVA, *P* > 0.05, Fig. 2A–F).

### Changes in α‐diversity indices are negatively correlated with changes in blood pressure

**Figure 3:**
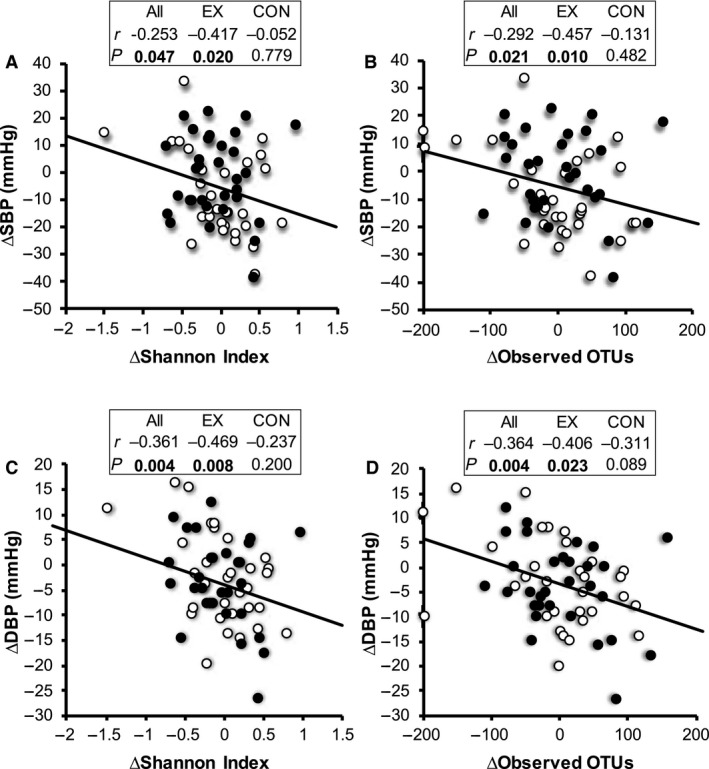
Association between changes in *α*‐diversity indices and changes in brachial blood pressure during intervention. Associations between (A) Shannon index and SBP, (B) observed OTUs and SBP, (C) Shannon index and DBP, (D) observed OTUs and DBP. Open circles represent changes in values during the exercise period; closed circles represent changes in values during control periods. Correlation was evaluated by Spearman's rank correlation coefficients. The level of statistical significance was set at *P* < 0.05.

Previous studies have demonstrated that gut microbiota diversity within individuals is associated with fitness/exercise (Clarke et al. 2014; Estaki et al. 2016), obesity (Turnbaugh et al. 2009; Le Chatelier et al. 2013), hypertension (Yang et al. 2015; Li et al. 2017), and various cardiometabolic risk factors (Le Chatelier et al. 2013; Kelly et al. 2016). Therefore, we examined the correlations between changes in *α*‐diversity indices and changes in CRF, obesity indices, and cardiometabolic risk factors during exercise intervention. Although the changes in Shannon diversity index and observed OTUs were not different between exercise and control periods, the changes in these indices were negatively correlated with the changes in SBP and DBP (Fig. 3A–D). These significant correlations were observed only during the exercise period regardless of the intervention order (Shannon diversity index and SBP: *r* = −0.417 *P* = 0.020; Shannon diversity index and DBP: *r* = −0.469 *P* = 0.008; observed OTUs and SBP: −0.457 *P* = 0.010; observed OTUs and DBP: *r* = −0.406 *P* = 0.023).

### Effects of endurance exercise on relative abundance of gut microbiota

**Figure 4:**
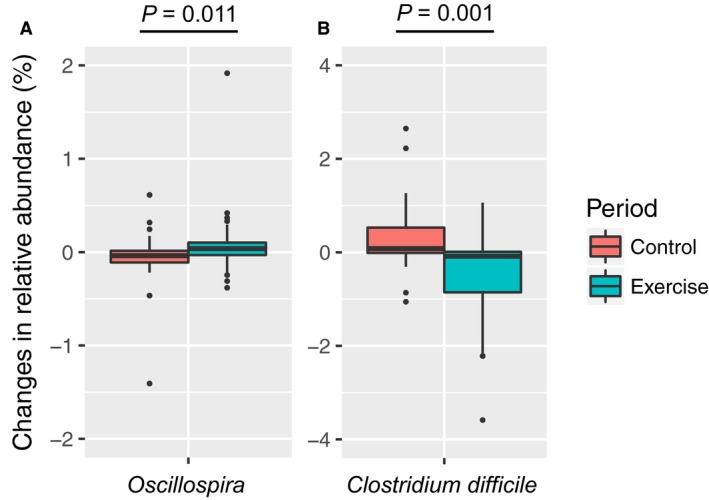
Comparison of changes in the relative abundance of (A) *Oscillospira and* (B) *Clostridium difficile* between exercise and control periods. Changes in the relative abundance of *Oscillospira and C. difficile* are presented as box‐plots with the median value and interquartile range. Significant differences were evaluated by the Mann–Whitney *U* test. The level of statistical significance was set at *P* < 0.05.

We examined the difference in the changes in the relative abundance of taxa between the exercise and control periods. There were no significant changes in the relative abundances of phylum, class, and order levels (data not shown). The relative abundance of the *Oscillospira* genus significantly increased during the exercise period as compared with those in the control period with no carryover effect with a post hoc statistical power at 41% (Fig. 4A). The treatment effect of *Oscillospira* was no longer significant after adjusting for changes in light‐colored vegetable, seaweed, and rice consumption (*P* = 0.236). An elderly subject was excluded from the comparison of *Oscillospira* because of abnormal changes, which exceeded 1%, in both the exercise and control period (Fig. 4A). The relative abundance of *Oscillospira* was significantly increased during the exercise period in the control first group (*P* = 0.003), whereas there was no significant change in the exercise first group (*P* = 0.88).

In addition, the relative abundance of *Clostridium difficile* significantly decreased at the species level with no carryover effect with a post hoc statistical power at 91% (Fig. 4B). The treatment effect for *C. difficile* remained significant after adjusting for changes in light‐colored vegetable, seaweed, and rice consumption (*P* = 0.035), which were foods that significantly changed throughout the intervention. The relative abundance of *C*. *difficile* was significantly reduced during the exercise periods in both the exercise first group (*P* = 0.03) and control first group (*P* = 0.01).

### Correlations between changes in *Oscillospira*,* C. difficile*, and cardiometabolic phenotypes

We examined the correlations between changes in *Oscillospira, C. difficile,* CRF, obesity indices, and cardiometabolic risk factors (Table 3). Data from the elderly individual who showed abnormal changes of *Oscillospira,* were excluded from the correlation analysis between *Oscillospira* and variables. Changes in the abundance of *Oscillospira* were significantly and positively correlated with changes in HDL cholesterol levels and negatively correlated with changes in HbA1c during the intervention and exercise period. Changes in *Oscillospira* were significantly and negatively correlated with changes in body fat percentage during intervention, although they did not change significantly during the exercise intervention.

**Table 3:** Correlations between changes in *Oscillospira* and *Clostridium difficile* composition and changes in cardiometabolic phenotypes

Periods	All		Exercise		Control	
	Rho	*P*	Rho	*P*	Rho	*P*
*Oscillospira* 1						
Body fat (%)	−0.333	**0.008**	−0.241	0.192	−0.429	**0.016**
*γ*−GTP (IU/L)	−0.120	0.352	−0.391	**0.030**	0.079	0.671
Total cholesterol (mg/dL)	0.252	**0.048**	0.176	0.343	0.145	0.438
HDL cholesterol (mg/dL)	0.334	**0.008**	0.500	**0.004**	−0.025	0.892
HbA1c (%)	−0.358	**0.004**	−0.474	**0.007**	−0.009	0.963
*Clostridium difficile*						
SBP (mmHg)	0.291	**0.022**	0.126	0.498	0.273	0.138
CAVI	0.306	**0.016**	0.047	0.803	0.301	0.100
$\dot{V}O_{2}\text{peak}$ (mL/kg/min)	−0.339	**0.007**	−0.088	0.639	0.007	0.970
Visceral fat area (cm^2^)	0.295	**0.020**	0.399	**0.026**	0.248	0.179
AST (IU/L)	0.365	**0.004**	0.274	0.135	0.345	0.058
ALT (IU/L)	0.309	**0.014**	0.248	0.178	0.392	**0.029**
Total cholesterol (mg/dL)	−0.363	**0.004**	−0.156	0.402	−0.337	0.064
HDL cholesterol (mg/dL)	−0.249	0.051	0.094	0.614	−0.378	**0.036**
LDL cholesterol (mg/dL)	−0.397	**0.001**	−0.248	0.178	−0.385	**0.032**
HbA1c (%)	0.305	**0.016**	0.150	0.419	0.272	0.139

Boldface indicates significance (*P* < 0.05). Spearman's rank correlation coefficients are shown. *N* = 31.

^1^ One participant was excluded from correlation analyses because of abnormal changes in *Oscillospira* during the exercise intervention period.

Changes in the abundance of *C. difficile* were significantly and positively correlated with changes in visceral fat area during the intervention and exercise period. Changes in *C. difficile* were significantly and positively correlated with changes in SBP, CAVI, AST, ALT, and HbA1c, and negatively correlated with changes in $\dot{V}O_{2}\text{peak}$, total cholesterol, and low density lipoprotein cholesterol during the intervention, although these variables did not change significantly during the exercise intervention (Table 3).

### Effects of endurance exercise on functional metagenomic profiles

**Figure 5:**
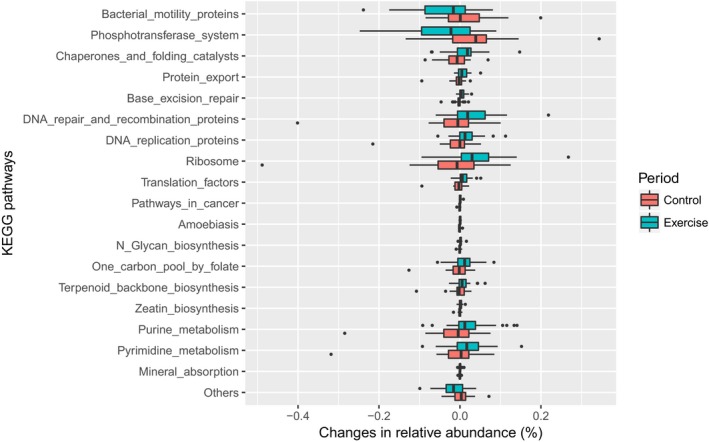
Changes in predicted metagenome functions that were significantly different between control and exercise periods. Changes in the relative abundance of each metagenomic function predicted based on the KEGG database are presented as box‐plots with the median value and the interquartile range. Significant differences were evaluated by the Mann–Whitney *U* test. All of the changes in metagenomic functions were significantly different between the exercise and control periods at the significance level of 0.05 and FDR < 0.3.

Lastly, we examined whether endurance exercise modulated specific metagenomic functions predicted by PICRUSt based on the KEGG database (Fig. 5). Although few members of the microbiota were modulated by 5‐week exercise at the taxonomic level, the changes in 19 metagenomic functions were significantly different between the exercise and control periods, with a post hoc statistical power at 43–85% (*P* < 0.05; FDR < 0.3). Furthermore, 15 of 19 metagenomic functions remained significant after adjusting for the changes in light‐colored vegetable, seaweed, and rice consumption (*P* < 0.05). Although significant differences between exercise and control periods were not observed if the subjects were stratified by the exercise first and control first groups and then separately analyzed, the direction of the exercise effect was the same between the exercise first and control first groups (data not shown). Particularly, the predicted metagenomic functions belonging to “Genetic Information Processing” (e.g., base excision repair, DNA repair and recombination proteins, DNA replication proteins, protein export, and ribosome and translation factors), and “Nucleotide Metabolism” (purine metabolism and pyrimidine metabolism) were overrepresented in the exercise period (Fig. 5).

## Discussion

We performed a 5‐week exercise intervention study to elucidate the effect of endurance exercise on microbiota composition and diversity in elderly men. While the 5‐week exercise program did not change the *α*‐diversity, the changes in *α*‐diversity during the interventions were negatively associated with changes in blood pressure. Furthermore, exercises increased the relative abundance of *Oscillospira* and decreased the abundance of *C. difficile*, which was also associated with improvements in cardiometabolic variables. These results suggest that short‐term endurance exercise has minor effects on gut microbiota diversity and composition in elderly individuals. Although independent effects of exercise should be discussed, the changes in the gut microbiota during the interventions were associated with cardiometabolic risks. Moreover, functional metagenomic profiles reveal some functional modulations of the gut microbiota during the exercise periods, providing preliminary insight into the role of exercise on gut metabolic functions.

It has been suggested that large interindividual differences in the gut microbiota exist even in healthy individuals (Human Microbiome Project C, 2012). Our *β*‐diversity analyses revealed that gut microbial communities were almost identical between pre‐ and postexercise fecal samples (Fig. 2A‐F). This suggests that endurance exercise does not play a large role in the overall variation of gut microbiota as compared to other factors such as host genetics and long‐term dietary patterns and environments. On the contrary, an elderly subject showed abnormal changes of gut microbial communities and was excluded from all analyses. It has been well‐documented that the gut microbiome can be affected by the use of antibiotics or dietary components (Chen et al. 2014; Voreades et al. 2014). The excluded subject may have unconsciously used these dietary components and/or antibiotics regardless of instruction.

Although the baseline $\dot{V}O_{2}\text{peak}$ was positively correlated with *α*‐diversity indices (Shannon index: *r* = 0.408, *P* = 0.025; observed OTUs: *r* = 0.411, *P* = 0.024), which was consistent with results of a previous study (Estaki et al. 2016), we did not detect an increase in *α*‐diversity during the exercise intervention (data not shown). Although *α*‐diversity indices were unaffected by exercise, increases in *α*‐diversity indices were significantly associated with decreases in SBP and DBP, particularly during the exercise periods (Fig. 3A–D). Interestingly, a recent cross‐sectional study demonstrated that middle‐aged hypertensive patients show reduced gut microbiota diversity as compared to body mass index‐matched healthy individuals (Li et al. 2017). Although we did not find any significant associations between baseline blood pressure levels and any *α*‐diversity indices (data not shown), our findings suggest that microbiota diversity is associated with intra‐individual variations in blood pressure. Although regular aerobic exercise is effective for prevention and treatment of hypertension, considerable interindividual variability in the ability to improve blood pressure in response to exercise has been described (Bouchard et al. 2012). The present study also showed that individuals demonstrate high variability in SBP and DBP changes, as well as *α*‐diversity indices, following exercise. It has been suggested that the responsiveness of the gut microbiota diversity to exercise is a predictor of exercise‐induced improvements in blood pressure. Recently, it was reported that fecal concentrations of short‐chain fatty acids (SCFAs) were increased by 6‐week endurance exercise in lean subjects (Allen et al. 2018), and it was suggested that the microbial SCFAs influence blood pressure by interacting with host SCFA receptors (Pluznick 2017). Although the fecal metabolites were not assessed in the present study, there is a possibility that exercise‐induced changes in SCFA production of gut microbiota were associated with the observed correlation between gut microbiota diversity indices and blood pressure.

The relative abundance of *Oscillospira* increased during the 5‐week exercise periods (Fig. 4A). *Oscillospira* is constantly detected by 16S ribosomal DNA sequence analysis in the human gut microbiome (Konikoff and Gophna 2016). Previous studies have suggested that the relative abundance of *Oscillospira* genus is positively associated with leanness (Tims et al. 2013; Goodrich et al. 2014), and is decreased in patients with Crohn's disease (Walters et al. 2014) and nonalcoholic steatohepatitis (Zhu et al. 2013). The results suggest that *Oscillospira* has a beneficial role on the host's health. The present study revealed that the changes in *Oscillospira* abundance were significantly correlated with the increase in HDL cholesterol levels and decrease in HbA1c during both the intervention and exercise periods (Table 3). The results suggest that the increase in *Oscillospira* during exercise periods may be associated with the host's cardiometabolic health. Since *Oscillospira* has resisted cultivation, which is impossible to use for probiotics, it is interesting and necessary as a further study to investigate the effects of prebiotics and/or fecal microbial transplantation on *Oscillospira*, and thereby to determine whether cardiometabolic disease is prevented. Although the correlation analysis also showed that the changes in *Oscillospira* genus were significantly correlated with body fat percentage during the intervention, significant associations were not observed during the exercise periods. Whether the relative abundance of *Oscillospira* affects the body composition must be evaluated in the future.

*Clostridium difficile* is a major cause of infectious diarrhea due to the production of toxins into the host gut (Kelly and LaMont 2008; Rupnik et al. 2009). *Clostridium difficile* infections have been epidemic worldwide, especially in hospitalized elderly patients (Collins et al. 2013; Burke and Lamont 2014; Lessa et al. 2015). To our knowledge, whether exercise status influences *C. difficile* composition is not known. Presently, 5 weeks of endurance exercise significantly reduced the relative abundance of *C. difficile* in the elderly (Fig. 4B). The result suggests that endurance exercise is beneficial for lowering toxin production by *C. difficile* in the gut. In addition, our correlation analysis showed that the changes in *C. difficile* abundance were associated with increases in $\dot{V}O_{2}\text{peak}$ and decreases in CAVI and HbA1c during the intervention (Table 3). However, there were no correlations between the variables during the exercise periods, suggesting that further study is necessary to examine whether a lower abundance of *C. difficile* is associated with changes in these variables. Changes in SBP, visceral fat area, AST, and ALT were also correlated with the changes in *C. difficile*. Although it is unknown whether the correlation was associated with exercise‐induced changes, it has been reported that obesity is a risk factor for *C. difficile* infection (Bishara et al. 2013; Leung et al. 2013). Therefore, it is plausible that the significant correlation between *C. difficile* and visceral fat area suggests that central obesity is a risk factor for *C. difficile* infection.

We also revealed that changes in several functional metagenomes predicted by PICRUSt were different between exercise and control periods. Interestingly, predicted metagenomic functions associated with “Genetic Information Processing” (e.g., base excision repair, DNA repair and recombination proteins, DNA replication proteins, protein export, ribosome, and translation factors), and “Nucleotide Metabolism” (purine metabolism and pyrimidine metabolism) were enriched for the exercise period, suggesting that endurance exercise enhances turnover of bacterial DNA and protein synthesis, which might be adaptations to gut environmental changes induced by exercise. In addition, it was reported that cafeteria‐fed obese rats have lower nucleotide metabolism (Kaakoush et al. 2017), and nucleotide metabolism pathway was reduced by chemotherapy, which leads to side effects, such as gastrointestinal mucositis (Montassier et al. 2015). The results suggest that exercise‐induced changes in metagenomic functions are opposite from obese and inflammatory microbiota function. Further studies are needed to examine whether the exercise‐induced metagenomic changes have beneficial roles for cardiometabolic and inflammatory risk factors.

The present study has several limitations. Since our study included only healthy male elderly subjects, it is still necessary to determine the effects of exercise on the gut microbiota in larger populations with both male and female individuals at varying ages and with different cardiometabolic conditions. Moreover, we did not control the diet during intervention although we assessed self‐reporting nutritional intake using a questionnaire; changes during intervention did not seem to influence the results of the present study. A diet‐controlled study is needed to determine the effects of the exercise training alone on the gut microbiome. The relatively short intervention period (5 weeks) is also a limitation. Because potential changes in the gut microbiota by regular exercise take months to years to manifest, long‐term effects of an exercise intervention on gut microbiota should be examined in the future. In addition, although we predicted functional metagenomic profiles based on 16S rRNA gene sequences, we did not directly analyze functional genes using shotgun metagenomic sequencing. Furthermore, we did not measure metabolites in fecal samples and plasma, which are produced by gut microbiota and which are thought to substantially affect the host phenotype. Shotgun metagenomic sequencing and comprehensive metabolome analysis may provide deep insight into the effects of exercise on microbial function and the underlying mechanisms for beneficial effects of exercise on the prevention of cardiometabolic diseases via gut microbiota and its metabolites.

To summarize, the present study employed a randomized crossover design to compare intra‐individual change in the gut microbiota. The results reveal that short‐term endurance exercise does not appreciably influence diversity and composition of gut microbiota, whereas minor changes in the gut microbiota are associated with cardiometabolic risk factors. In addition, our preliminary analysis suggests some changes in metagenomic function during exercise periods. The findings of this study provide realistic and novel functional insights into the gut microbiome, and highlight the gut microbiota as a mediator of cardiometabolic benefits. Further large‐scale and long‐term studies are needed to elucidate the relationship between exercise and gut microbiota, and to reveal beneficial roles of the gut microbiome in the prevention of cardiometabolic diseases.

## Conflict of Interest

The authors declare that they have no competing interests.
